# MicroRNAs Show Mutually Exclusive Expression Patterns in the Brain of Adult Male Rats

**DOI:** 10.1371/journal.pone.0007225

**Published:** 2009-10-06

**Authors:** Line Olsen, Mikkel Klausen, Lone Helboe, Finn Cilius Nielsen, Thomas Werge

**Affiliations:** 1 Institute for Biological Psychiatry, Psychiatric Centre Sct. Hans, Roskilde, Denmark; 2 Department of Clinical Biochemistry, Copenhagen University Hospital, Rigshospitalet, Copenhagen, Denmark; 3 Discovery Biology Research, H. Lundbeck A/S, Copenhagen, Denmark; Victor Chang Cardiac Research Institute (VCCRI), Australia

## Abstract

**Background:**

The brain is a major site of microRNA (miRNA) gene expression, but the spatial expression patterns of miRNAs within the brain have not yet been fully covered.

**Methodology/Principal Findings:**

We have characterized the regional expression profiles of miRNAs in five distinct regions of the adult rat brain: amygdala, cerebellum, hippocampus, hypothalamus and substantia nigra. Microarray profiling uncovered 48 miRNAs displaying more than three-fold enrichment between two or more brain regions. Notably, we found reciprocal expression profiles for a subset of the miRNAs predominantly found (> ten times) in either the cerebellum (miR-206 and miR-497) or the forebrain regions (miR-132, miR-212, miR-221 and miR-222).

**Conclusions/Significance:**

The results indicate that some miRNAs could be important for area-specific functions in the brain. Our data, combined with previous studies in mice, provides additional guidance for future investigations of miRNA functions in the brain.

## Introduction

MicroRNAs (miRNAs) are a group of small 19–24 nt non-coding RNAs that control protein levels through inhibition of mRNA translation or stability [1], [2]. Over-expression or knock-down of individual miRNAs has shown that miRNAs can affect the mRNA levels of many genes [2]–[4]. It has therefore been suggested that a miRNAs may affect several cellular processes and be involved in punctual control of the cellular state and the precision of developmental processes [5].

A relatively large number of the known miRNAs are expressed in the mammalian brain [6], [7], but very little is known about the functions regulated by these brain-expressed miRNAs. Some studies have shown an involvement of miRNAs (e.g. miR-9a and miR-124) in neuronal development and differentiation [8]–[10]. Conditional deficiency of the miRNA maturation enzyme Dicer in the developing mouse telencephalon leads to postnatal death due to hypotrophy of neurons and neurogenic progenitors, whereas Dicer ablation has a far smaller effect in the neural stem-cells [11]. Likewise, disruption of Dicer is also associated with the degeneration of cerebellar Purkinje cells and the development of ataxia in mice [12]. miR-132 has also been related to neuronal morphogenesis [13], [14], and both miR-132 and miR-219 have been shown to modulate the circadian clock [15]. Other studies have indicated that some miRNAs are involved in synapse functioning [16]–[18].

Interestingly, several miRNAs have been shown to be involved in neurological and psychiatric diseases. We have previously shown associations between schizophrenia and SNPs located in the vicinity of the mir-206/mir-133b cluster and mir-198 [19]. Furthermore, miR-133b is involved in the maturation and function of the midbrain dopaminergic neurons that malfunction in patients with Parkinson's disease [15], [20]. Recently, miR-219 has been shown to target calcium/calmodulin-dependent protein kinase II gamma subunit [21], which is involved in N-methyl-d-aspartate glutamate receptor-mediated signalling and implicated in schizophrenia. Moreover, the mir-175 locus is localized within the candidate region for the Waisman syndrome (early-onset parkinsonism) and X-linked mental retardation [22]. Finally, the DGCR8 gene positioned at chromosome 22q11, which is a strong susceptibility region for schizophrenia, is involved in miRNA maturation [23]. Thus, the presence of miRNAs in adult brain tissues and their association with brain dysfunctions indicate that some miRNAs are involved in maintaining the functions of the brain, not only during development, but also throughout life [24].

The brain is the most complex tissue in the mammalian organism, and it shows both regional and left-right subdivision in function and anatomy in many structures. This is well-known in humans [25], but there is accumulating evidence of functional asymmetries and associated anatomical lateralization of the left and right brain hemispheres also among non-primates within the vertebrate lineages (amphibians, birds, fish, mammals and reptiles) [26]–[28]. At the molecular level, hemispheric asymmetry in mRNA expression has been reported both between the left and right hippocampus in rats [29] and in the developing human and mouse cortex [30].

The functions of brain-expressed miRNA have been investigated in recent years, but the detailed mapping of miRNA expression patterns in the brain is sparse and the functions of most of the miRNAs in normal brain tissue still remain to be elucidated. Their regulatory properties, the pleiotrophic effects they are assumed to have, and their high abundance in the brain indicate that miRNAs may be involved in area-specific functions of the adult rat brain. So we investigated whether brain-expressed miRNAs are equally abundant in various brain regions in unstimulated male rats.

## Materials and Methods

### 2.1 Samples

Adult male Sprague-Dawley rats (obtained from Taconic, Denmark) were sacrificed by decapitation. The brain was quickly removed and samples were immediately dissected on a cold surface, snap-frozen in pulverized dry ice, and stored at −80°C until further processing. For each animal, 3–4 brain regions were dissected from the right and left hemispheres separately. In total, samples from 6 different brain regions were obtained and each brain region was sampled from a total of 4 animals.

The rat brain stereotaxic atlas was used for identification and delineation of brain regions (Paxinos and Watson) [31]. The hippocampus was exposed by removal of the cortex and the entire hippocampal structure was dissected out. The prefrontal cortex was dissected from a 1 mm slice (centered around Bregma 3.2 mm). An area delineated by the upper and lower part of the forceps minor corpus callosum was dissected from the slice. The hypothalamus and the amygdala were sectioned from a 1 mm slice centered around Bregma −3.14 mm. We used the internal capsule and the mammillothalamic tract to delineate the hypothalamus. We used the mammillothalamic tract, the piriform cortex and the optic tract to delineate the amygdaloid region (AM). An area of the ventral mesencephalon enriched with substantia nigra was dissected from a ∼0.8-mm slice centered around Bregma −5.20 mm. This region was delineated by the horizontal midline of the mesencephalon and the lateral border of the mesencephalon. The medial third of the slice was removed (with section lines parallel with the dorsoventral midline). The cerebellum was dissected as a whole.

All animals were handled in strict accordance with good animal practice under licence from the Danish Animal Experimentation Inspectorate and following the guidelines of the European Communities Council Directive of 24 November 1986 (86/609/EEC).

### 2.2 RNA isolation

Frozen samples were quickly homogenized in QIAzol Lysis Reagent (Qiagen, Germany) and the <200 nt RNA fraction (including miRNAs) was immediately extracted using the RNeasy® Lipid Tissue Mini Kit (Qiagen, Germany) following the modified protocol which enables purification of miRNA and total RNA from the same sample in subsequent steps.

### 2.3 miRNA array hybridization and analysis

The NCode™ Multi-Species miRNA Microarray dual-colour system V2 (Invitrogen, Carlsbad, CA, USA) was used for miRNA expression analysis. These miRNA microarrays can detect all known mature miRNAs in mirBase 9.0 and include specific probes for 236 known rat miRNAs. Each probe is spotted in triplicate on each array. All samples were hybridized against a human brain universal reference RNA sample (AM6051, Applied Biosystems, Foster City, CA, USA). This design allowed for comparison between hemispheres as well as regions. Each miRNA sample (600 ng) was poly-A tailed and tagged with the sequence tag for Alexa Fluor® 5 fluorophor using the NCode™ miRNA Labeling System (Invitrogen, Carlsbad, CA, USA), while the universal reference RNA was labelled using Alexa Fluor® 3. Samples were hybridized against the reference to NCode™ Multi-Species miRNA Microarrays (Invitrogen, Carlsbad, CA, USA) overnight using the NCode™ Multi-Species miRNA Microarrays Kit (Invitrogen, Carlsbad, CA, USA) and following the instructions given by the manufacturer. Arrays were then hybridized with Alexa Fluor® 3 and Alexa Fluor® 5 capture reagents and washed. Hybridization was performed on a MAUI hybridization station (BioMicro Systems, Inc, Salt Lake City, UT, USA). Each array was subsequently scanned using an Agilent DNA microarray scanner, and images were processed using the GenePix Pro software (Molecular Devices, Sunnyvale, CA, USA).

Data analyses were conducted using the freely available statistical program “R” and several packages from the Bioconductor project. The LIMMA package [32]–[34] was used for normalization and differential expression analysis of the microarray data. Raw data were background-adjusted using the “normexp” correction method with an offset of 50. Print-tip-loess normalization was subsequently used to adjust data within each array as recommended by Hua et al [35] for miRNA cDNA arrays. To allow for comparisons between arrays, we used Gquantile normalization. The Gquantile procedure forces the intensities obtained from the green channel (the signal from the universal reference) to the same value for all the arrays and adjusts the signal from the red channel (the samples under investigation) accordingly. The signals from replicate spots within each array were averaged for the subset of probes that are specific for rats (n = 236) and a linear model was fitted for each of these rat-specific miRNAs. The Benjamini and Hochberg (BH) method was used to control the false discovery rate [36] and thereby adjust for multiple testing of several miRNAs at the same time. A transcript was considered present if the probe signal was at least double that of the background, which corresponds to a raw signal of 100 units. The data has been deposited in NCBI's Gene Expression Omnibus [37] with the accession number GSE16725 and can be accessed at http://www.ncbi.nlm.nih.gov/geo/query/acc.cgi?acc=GSE16725. The within-region variability of selected miRNAs were calculated as the standard deviation (sd) of the fully normalized data and the F-test was used to test for equal variances between individual miRNA and the mean sd of all miRNA within a region.

To examine the quality of the expression data we performed unsupervised hierarchical clustering on the fully normalized expression data using the “R” package's “cluster”, “bioDist” and “Mfuzz” [38] with options for Spearman distribution and Ward clustering. The branch lengths of a cluster dendrogram are measures of the distance between the samples and thus a visual presentation of the variability between samples within and between regions. Heat maps were used to provide a visual representation of the relative differences between the various brain regions (regardless of hemisphere) and were generated in “R” using the “gplots”, “RColorBrewer” and “fields” packages. The heat maps are based on the results obtained from the cluster analysis and only miRNA genes that showed statistically significant differences (p<0.01) in expression between two or more regions after correction for multiple testing by BH and had an absolute log2 fold-change greater than 10 or a log2 fold-change larger than 3 are represented in the figures.

### 2.4 qRT-PCR evaluations of microarray results

For some microRNAs (specified below), a TaqMan® MicroRNA Assay from Applied Biosystems (AB, Foster City, CA, USA) was used for qRT-PCR evaluation of the results of the microarray analyses. The qRT-PCR reactions were performed on the same RNA samples that were applied to the microarrays. Normalization was done with miR-103 (part # 4373158) as an endogenous control and the abundance of each miRNA was determined using the relative standard curve method. The selected miRNAs were rno-let-7a (part # 4373169), rno-miR-132 (part # 4373143), rno-miR-206 (part # 4373092) and rno-miR-320 (part # 4395388). Reverse transcription was carried out in triplicate with the TaqMan® MicroRNA RT Kit (AB, Foster City, CA, USA) using the manufacturer's recommended protocol on each of the 4 biological replicates from the relevant regions and hemispheres (see Table 1). Real-time PCR was performed in duplicate on an iCyler real-time PCR instrument (Bio Rad Laboratories Inc, Dr. Hercules, CA, USA) using 2× Universal PCR Master Mix, no AmpErase UNG (AB, Foster City, CA, USA). The endogenous reference (miR-103) was chosen from the microarray expression data set on the basis of the following criteria: uniform and relatively high expression levels in all brain regions and hemispheres. Differences between the two hemispheres were calculated as a ratio of the average miRNA abundance in the right and left hemispheres respectively, and two-tailed t-tests were used to test the hypothesis if there were differences between the right and left-hand sides of each region.

**Table 1:** qRT-PCR failed to replicate array data on right/left miRNAs asymmetry in the brain.

		Array data			qRT-PCR data	
miRNA	Region	R/L FC	P	Adj. P	R/L ratio	P
let-7a	hip	0.67	0.007	0.52	1.08	0.95
	hyp	1.70	0.0003	0.04	0.71	0.73
miR-132	hip	0.73	0.02	0.78	0.99	0.68
	hyp	1.39	0.01	0.48	0.91	0.62
miR -320	hip	0.66	0.005	0.52	1.16	0.52
	hyp	1.42	0.02	0.66	0.95	0.42
miR -448	cb	0.46	0.03	1.00	nd	nd
	hip	2.18	0.04	0.96	nd	nd
miR -497	cb	1.61	0.02	1.00	nd	nd
	hip	1.60	0.02	0.96	nd	nd

cb: cerebellum; hip: hippocampus; hyp: hypothalamus; R/L FC: Fold change between the right and left hemisphere of the given region; P: nominal P-value; Adj. P: Adjusted P-value corrected for multiple testing by BH; R/L ratio: Ratio of the relative miRNA expression levels in the right and left hemispheres, nd: not done.

## Results

### miRNA profiling in brain regions

Tissue-specific expression patterns can provide important clues to the physiological function of a miRNA. Comprehensive studies on miRNA expression throughout the mouse brain have been published [39], [40], but comparable studies in rats are lacking. Using miRNA gene expression arrays, we measured the expression of a large set (n = 236 miRNA) of known miRNAs across six regions of the rat brain. This expression set represents the normal miRNA expression profile and allowed us to examine global trends in miRNA gene expression in the brain of adult male rats.

**Figure 1:**
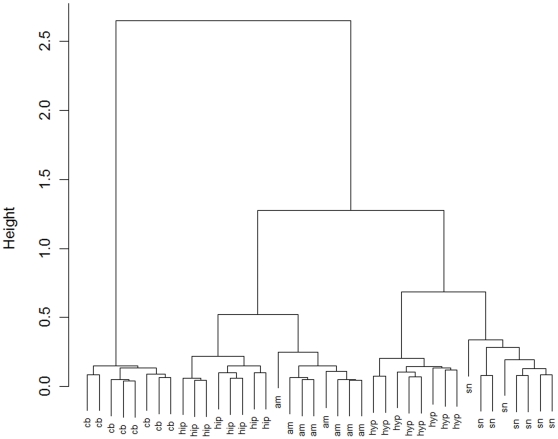
Cluster dendrogram of miRNA expression profiles demonstrates samples grouped according to their biological origin in the brain of adult male rats. The dendrogram shows that the samples from five brain regions are grouped according to their biological relatedness, suggesting that the miRNA expression profiles overall contain region-specific information. The branch lengths of the dendrogram are measures of the difference between samples and show that the within-region variability in miRNA abundances is low compared to the between-region variability. Average standard deviations of the regional miRNA expression levels are 0.43 (am), 0.37 (cb), 0.39 (hip), 0.43 (hyp) and 0.47 (sn). am: amygdala; cb: cerebellum; hip: hippocampus; hyp: hypothalamus; sn: substantia nigra.

We used hierarchical clustering to examine the quality of the data from all six brain regions. Samples obtained from the individual regions were grouped together into clusters that reflected their biological relationship (Figure 1 and Figure S1). The variability between the brain regions (measured by the height of the dendrogram branches) is considerably larger than the variability within the regions. This suggests that the overall expression patterns of the miRNA have a tissue-specific signature. One exception from this pattern was a subset of the samples obtained from the prefrontal cortex, which were grouped within the amygdaloid and hippocampus clusters (Figure S1). However, the sample from the amygdala and the hippocampus were clearly different and separated into biologically meaningful groups. The prefrontal cortex is small in rats and samples from this region may therefore be enriched with tissue from surrounding areas. In view of the diffused clustering of the samples obtained from the prefrontal cortex, all samples from this region were excluded from subsequent analyses. When data from the prefrontal cortex were omitted, we found that the miRNA expression profiles accurately distinguished the various regions of the brain (Figure 1). Most pronounced was the cerebellum, which clustered separately from the four regions in the forebrain.

**Figure 2:**
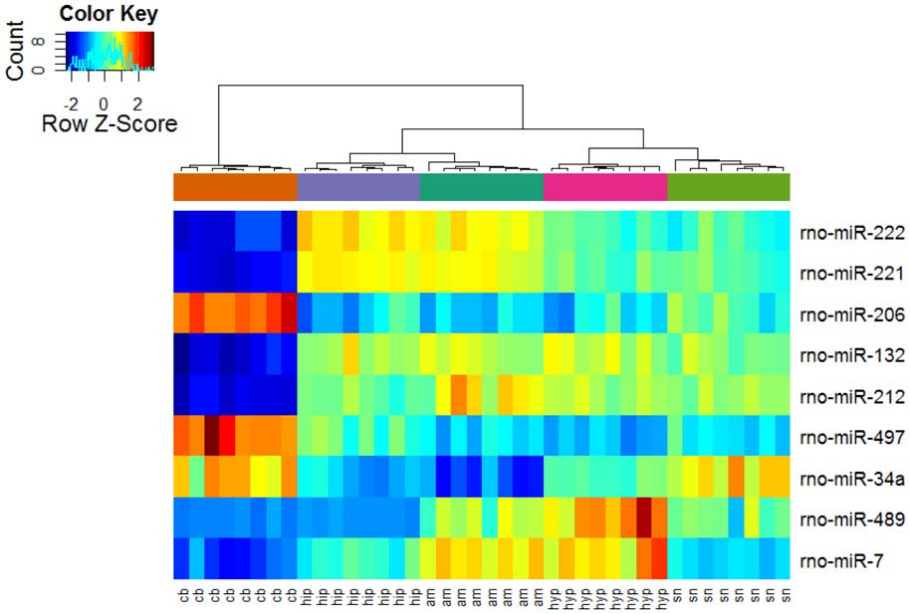
Heat map of miRNAs, displaying more than ten-fold region-specific enrichment between regions in the adult male rat brain. Overview of the miRNA genes that show statistically significant differences (p = 0.01) in expression levels between two or more regions (after correction for multiple testing by BH) and have an absolute log2 fold-change greater than 10. The abundance of individual miRNA in each sample is depicted using a colour code, where relatively high or low abundances of transcripts are shown in red and blue respectively. am: amygdala; cb: cerebellum; hip: hippocampus; hyp: hypothalamus; sn: substantia nigra.

Most of the miRNA on the array was detected in all samples across all regions (mean = 181 miRNAs) and in a majority of cases only subtle differences were seen between the different regions. Only 15.3% (36 miRNAs) of the miRNAs had very low abundances in all regions (with probe signals less than twice the background). In line with studies in mice and zebra fish, we found that especially miR-124a and miR-29a were highly abundant in all regions, whereas the expression levels for miR-9 were more moderate (data not shown) [7], [41]–[45]. Strong region-specific expression patterns were seen for a minor set of nine miRNAs, which showed more than a ten-fold change (p<0.01) in abundances between regions (Figure 2). Although the relative abundances of these nine miRNAs were low (i.e. compared to miR-124a), the transcripts were well above the background levels. The most pronounced differences in expression patterns among these nine miRNAs were seen for the miR-221 family members (miR-221 and miR-222), which showed a cerebellar reduction of more than 60-fold compared to the hippocampus and the amygdaloid region. Forebrain enrichment was also seen for the two members of the miR-132 family (miR-132 and miR-212), which were also most highly expressed in the hippocampus and amygdaloid regions. Inversely, miR-206 and miR-497 were relatively more abundant (>10 times) in the cerebellum compared with the various forebrain regions, whereas the hypothalamus clustered separately from the other regions due to the expression of miR-489, which was nearly absent in the cerebellum and the hippocampus. The diversity in miRNA expression patterns is depicted in Figure S2, which shows miRNAs with more than three-fold region-specific enrichment between any two regions of the brain. In all, we found moderate (three-fold) region-specific enrichment of 48 miRNAs (e.g. miR-138, miR-195 and miR-218, Figure S2).

miR-206 has previously been classified as a muscle-specific miRNA [46] that is localized to the neuro-muscular junctions and it has been shown to be regulated by neuronal stimulation of the muscle fiber [47]. Thus, the presence of miR-206 in the cerebellum was intriguing and we used qRT-PCR to verify the presence of this miRNA in the cerebellum, the amygdala and the hippocampus. The qRT-PCR results showed good correlation to data from the array analysis with miR-206 being 27× and 39× more abundant in the cerebellum than in the amygdala and the hippocampus respectively.

### Analyses of asymmetric expression of miRNA in the brain

To investigate whether miRNA expression levels differed between the two hemispheres, we compared miRNA abundances of the right and left side of the brain within each region. As shown in Table 1, nominal differences between the right and left hemispheres were found for five miRNAs in two regions. However, the differences were subtle (fold-change less than 2) and only data for one miRNA (let-7a) were resistant to correction for multiple testing in one region (the hypothalamus). The within-region variability of miR-132 (sd = 0.38 and 0.37), miR-320 (sd = 0.38 and 0.44), miR-497 (sd = 0.47 and 0.27) and let-7a (sd = 0.40 and 0.55) did not differ from the regional average of the hippocampus (sd = 0.39) and the hypothalamus (sd = 0.43) respectively (P-values from 0.13 to 0.95). However, the variability of miR-448 in the cerebellum (sd = 1.58) and the hippocampus (sd = 1.29) did differ from the regional mean (P<0.01).

To examine whether the findings from the array data represented true right/left differences, we used qRT–PCR to measure the relative abundance of let-7a, miR-132 and miR-320. We found no evidence for hemispheric differences in gene expression levels for either of the miRNAs investigated (Table 1), which suggests that the minor right/left differences seen in the array data represent false positive findings.

## Discussion

We have characterized the miRNA expression profiles in both hemispheres of five anatomically distinct brain regions, thus providing an overview of the miRNA present in the rat brain. Overall the brain tissues were characterized by having measurable levels of the majority of the known rat miRNAs. This is consistent with data on mouse brain tissues published previously [6], [7], [43].

### Differentially expressed miRNAs in five brain regions

Our data provides a detailed map of miRNA brain expression in rats and shows that there are some differences in the expression in the cerebellum of a subset of the detectable transcripts, which are either highly enriched (miR-206 and miR-497) or nearly depleted (miR-132, miR-212, miR-221 and miR-222). Accumulations of miR-497 in the cerebellum, of miR-7 in the hypothalamus, and of miR-221 and miR-222 in the hippocampus have also been described in mice [39] and zebrafish (larval and adult brain), where miR-222 is expressed in specific groups of differentiating cells in the rostral parts of the brain [42]. Moreover, the expression profile for miR-34a overlaps with that reported in zebrafish [42]. In line with previous studies in mice, we also found moderate enrichment of miR-195 [39], [40] in the cerebellum and of miR-218 in the hippocampus [39] (Figure S2).

In the light of the anatomical and functional differences between brain regions, miRNA region-specific expression may not be surprising. However, it is intriguing that the expression of a subset of the miRNAs appears to be mutually exclusive between different parts of the brain. The cerebellum is mainly involved in controlling movement and coordination and the neuronal circuits in the cerebellar cortex are conserved across most of the vertebrate lineages [48]. So the distinction of the cerebellum from the forebrain regions, as seen in the miRNA expression profiles, may reflect this evolutionary conservation and suggest that some miRNAs regulate either cerebellar or some forebrain-specific functions. Indeed, the overlapping expression patterns of miR-222 in zebrafish, mice and rats suggest that this miRNA is important for some specialized hippocampal functions.

### Suggested functions of miRNAs with regional expression profiles

Some clues to the function of the individual miRNAs may be deduced from information on their target genes. Most of the known validated targets for the nine miRNAs that show large regional differences in their abundances in the rat brain (highlighted in Figure 2) have been found in tissues and cells outside the central nervous system. Still, some known miRNA-mRNA relationships may be worth attention, although precautions should be taken when extrapolating findings from somatic cells to the brain.

### *miRNA boundaries: the control of converging pathways?*

Some of the miRNAs that we found to be expressed in a mutually exclusive way between the different brain regions are regulators of proteins involved in the same molecular processes or even sharing the same mRNA targets. This suggests that there is an overlapping functional relationship between some of these miRNAs.

One example is a potential functional cross-talk between miR-206 (enriched in the cerebellum) and miR-7 (enriched in the hypothalamus and amygdala). It has recently been shown that the insulin-like growth factor 1 (IGF-1) is targeted by miR-206 [49] and that miR-7 is a repressor of insulin receptor substrate 1 (IRS1) and 2 (IRS2) [50]. IGF-1 is the cognate substrate for the insulin-like growth factor 1 receptor (IGF-1R) and receptor binding of IGF-1 induces autophosphorylation and the recruitment of IGF-1R adaptor proteins such as IRS1 and IRS2 to initiate the intracellular signal transduction. IGF-1 and IGF-1R are both expressed throughout the brain in neurons, astrocytes and in the vasculature, but circulating IGF-1 also crosses the blood-brain barrier with high affinity. The close interdependence between the actions of IGF-1 and IRS1/IRS2 suggests that miR-206 and miR-7 could be involved in tissue or cell-specific regulation of the functions mediated by IGF-1-signalling pathways in the brain.

In the brain, the actions of IGF-1 are modulated by estrogen receptor alpha (ESR1), which directly interacts with several components of the IGF-1 transduction pathway including IGF-1R and IRS1 [51]. Interestingly, in vitro studies in breast cancer cell lines have demonstrated that miR-206 [52]–[54] represses the translation of ESR1 mRNA. As such, the translational regulation of ESR1 represents another remarkable functional cross-talk between miRNAs, which we show to be expressed in a mutually exclusive manner between the cerebellum (miR-206) and the hippocampus/amygdaloid regions (miR-221/miR-222).

Strikingly, two of the downstream targets of IGF-1 signalling, namely connexin 43 and zona occludens 1, are also validated targets for miR-206 [46], [55] and miR-212 [56], [57] (depleted from the cerebellum) respectively. Furthermore, miR-34a (up-regulated in the cerebellum and the substantia nigra) may modulate the cellular response to IGF-1 through the regulation of vascular endothelial growth factor A, which is targeted by miR-34a [58].

The examples outlined indicate that several of the miRNAs that show mutually exclusive expression patterns in the brain have a potential functional overlap that converge onto IGF-1 signalling pathways and downstream targets. Normal brain development and function depend on IGF-1 signalling in spatial and temporal-specific patterns, and IGF-1 acts as an autocrine or paracrine factor to promote proliferation of neuronal progenitors, neuronal and oligodendrocyte differentiation and survival [59]. Moreover, IGF-1 stimulates glucose uptake by nerve terminals, homeostasis, and anabolic processes, and it is also strongly induced in astrocytes in response to central nervous system injury [60].

Multiple levels of regulation may be necessary to shift the action of IGF-1 toward the required function. The required functions may depend on the micro-environmental conditions, cell type, or subcellular localization, and the differential expression of regulatory miRNAs may facilitate the process for obtaining strict tissue-specific and timely control of such functions.

## Supporting Information

Figure S1 Cluster analysis demonstrating miRNAs grouped according to their regional expression profiles in adult male rat brain including samples from the prefrontal cortex. The dendrogram shows that the samples from six brain regions are grouped according to their biological relatedness except samples from prefrontal cortex (pfc). The branch lengths of the dendrogram are measures of the difference between samples and show that the within-region variability in miRNA abundances is low compared to the between-region variability. am: amygdala; cb: cerebellum; hip: hippocampus; hyp: hypothalamus; sn: substantia nigra.
(2.39 MB TIF)

Figure S2 Heat map highlighting genes showing more than threefold difference in miRNA abundances between regions in adult male rat brain. Genes that showed statistical significant differences (p = 0.01) in expression between two or more regions after correction for multiple testing by BH and had an absolute log2-fold-change greater than 3 are represented. am: amygdala; cb: cerebellum; hip: hippocampus; hyp: hypothalamus and sn: substantia nigra.
(2.39 MB TIF)
