# Supplementary figures and images for: MicroRNAs Show Mutually Exclusive Expression Patterns in the Brain of Adult Male Rats
Source: PLoS One. 2009 Oct 6;4(10):e7225. doi: 10.1371/journal.pone.0007225 (PMC2752988; doi:10.1371/journal.pone.0007225)

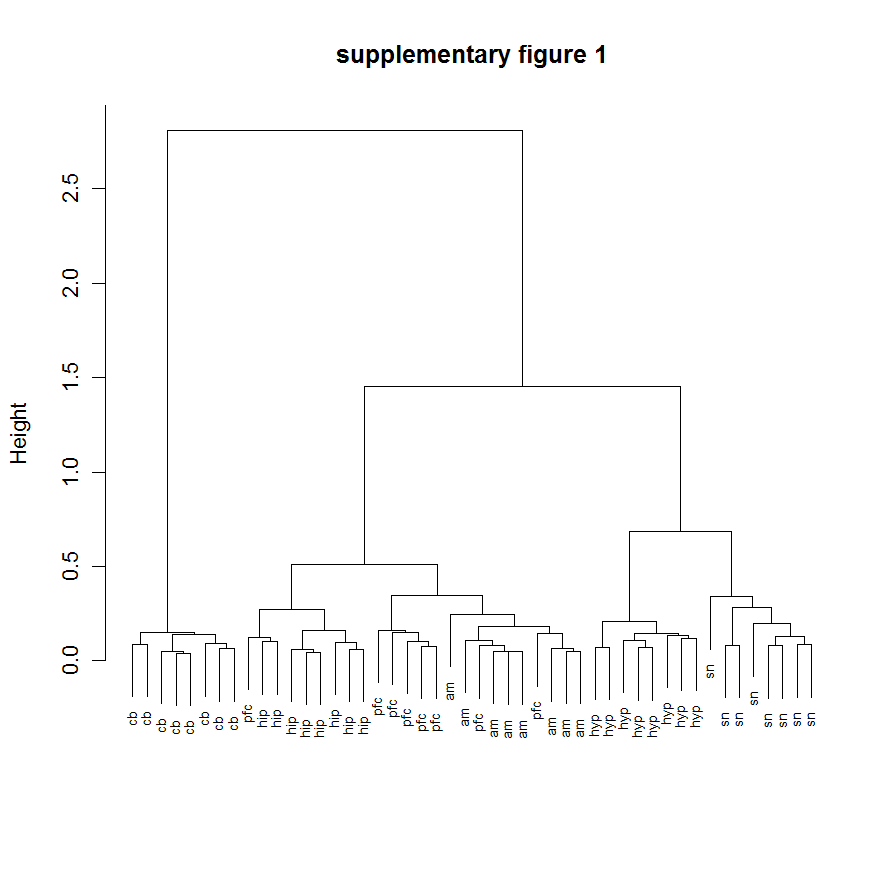

Supplement: Figure S1 — Cluster analysis demonstrating miRNAs grouped according to their regional expression profiles in adult male rat brain including samples from the prefrontal cortex. The dendrogram shows that the samples from six brain regions are grouped according to their biological relatedness except samples from prefrontal cortex (pfc). The branch lengths of the dendrogram are measures of the difference between samples and show that the within-region variability in miRNA abundances is low compared to the between-region variability. am: amygdala; cb: cerebellum; hip: hippocampus; hyp: hypothalamus; sn: substantia nigra. (2.39 MB TIF) [file pone.0007225.s001.tif]

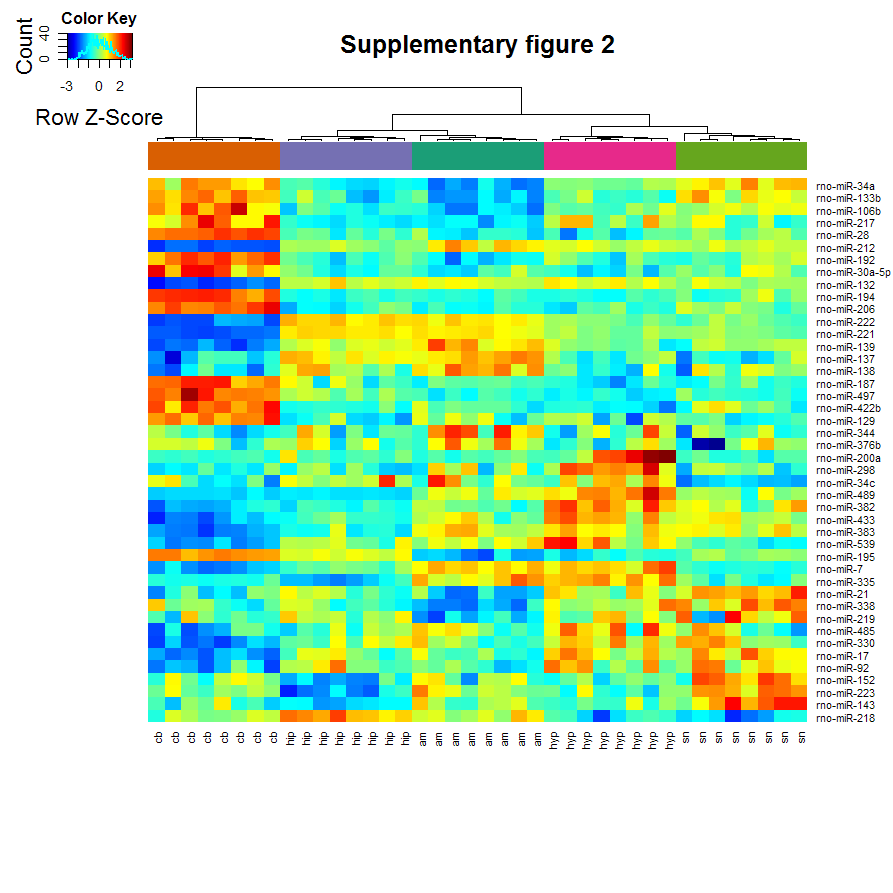

Supplement: Figure S2 — Heat map highlighting genes showing more than threefold difference in miRNA abundances between regions in adult male rat brain. Genes that showed statistical significant differences (p = 0.01) in expression between two or more regions after correction for multiple testing by BH and had an absolute log2-fold-change greater than 3 are represented. am: amygdala; cb: cerebellum; hip: hippocampus; hyp: hypothalamus and sn: substantia nigra. (2.39 MB TIF) [file pone.0007225.s002.tif]
